# Implementing a tracking system for confirmatory diagnostic results after positive newborn screening for cystic fibrosis—implications for process quality and patient care

**DOI:** 10.1007/s00431-020-03849-4

**Published:** 2020-10-26

**Authors:** Gwendolyn Gramer, Inken Brockow, Christiane Labitzke, Junmin Fang-Hoffmann, Andreas Beivers, Patrik Feyh, Georg F. Hoffmann, Uta Nennstiel, Olaf Sommerburg

**Affiliations:** 1 Center for Pediatric and Adolescent Medicine, Department of General Pediatrics, Division of Neuropediatrics and Metabolic Medicine, University Hospital Heidelberg, Im Neuenheimer Feld 430, 69120 Heidelberg, Germany; 2 Screening Center, Bavarian Health and Food Safety Authority (LGL), Veterinaerstrasse 2, 85764 Oberschleissheim, Germany; 3 Translational Lung Research Center (TLRC), German Lung Research Center (DZL), University of Heidelberg, Heidelberg, Germany; 4 University of Applied Sciences (Hochschule) Fresenius, Infanteriestraße 11a, 80797 Munich, Germany; 5 Center for Pediatric and Adolescent Medicine, Division of Pediatric Pulmonology & Allergy and Cystic Fibrosis Center, University Hospital Heidelberg, Im Neuenheimer Feld 430, 69120 Heidelberg, Germany

**Keywords:** Prevention, Cystic fibrosis, Screening, Tracking, Process quality

## Abstract

Newborn screening for cystic fibrosis (CF-NBS) was introduced in Germany in 2016. Currently, systematic follow-up of positive CF-NBS results is not implemented or reimbursed in the NBS program. We investigated results of confirmatory testing over 24 months after implementation of CF-NBS for a large German NBS center before and after introduction of an active tracking system and performed a cost calculation for tracking. Results are compared with the federal state of Bavaria, where a centralized tracking system has been in place for many years. At the NBS center, 244 of 281,907 children had a positive CF-NBS result requiring diagnostic confirmation. Before implementation of a telephone tracking system, only 43% of confirmatory results were returned despite repeated written requests. The consecutive strategy including telephone tracking led to an increase of resolved cases to 84%. However, the centralized tracking system in Bavaria, assigning children with positive CF-NBS directly to a responsible CF-center, resolved 99% of cases. The calculated additional cost for a tracking system in Germany including telephone tracking is 1.20€ per newborn screened.

*Conclusion*: The implementation of a tracking system achieves a distinct improvement in CF-NBS with justifiable costs. The effect can be limited by absence of centralized organization of confirmatory testing.

**Table:** 

**What is Known:** *• Newborn screening for cystic fibrosis (CF-NBS) has been performed for many years in several countries worldwide* *• While many studies have focused on different CF-NBS strategies, the organization of confirmatory testing and process quality concerning returned information to the NBS center has so far received less attention.*	
**What is New:** *• The implementation of an active tracking system achieves a distinct improvement of clarified cases after positive CF-NBS with justifiable costs.* *• The effect of a tracking system can be limited by the absence of a centralized organization of confirmatory testing.*

**Supplementary Information:**

The online version contains supplementary material available at 10.1007/s00431-020-03849-4.

## Introduction

Newborn screening (NBS) is the most successful measure of secondary prevention in medicine [1, 2]. NBS for cystic fibrosis (CF-NBS) has been performed in many countries worldwide for several decades [3] and was introduced into the German national NBS panel in September 2016 [4]. NBS in Germany is regulated by the Pediatrics Directive of the Federal Joint Committee (Gemeinsamer Bundesausschuss, G-BA) [5]. This includes the NBS strategy and the flow of information after positive NBS results. The algorithm of CF-NBS currently used in Germany is a three-step protocol based on measurement of immunoreactive trypsin (IRT) as the primary marker (Supplementary Figure 1). It includes a second-tier and third-tier approach measuring pancreatitis-associated protein (PAP) and, if positive, 31 *CFTR* mutations. In cases of ultrahigh IRT (> 99.9 percentile), CF-NBS is directly classified as positive (“safety net strategy”, SN). After positive CF-NBS, diagnostic confirmation includes measurement of sweat chloride (sweat test) and clinical assessment [5, 6]. The CF-NBS protocol in Germany results in a relatively high number of false-positive results, especially via the SN strategy due to unspecific IRT elevations [7]. A CF diagnosis is confirmed in only one in five infants screened positively for CF [7–9].

According to the Pediatrics Directive of the G-BA, positive CF-NBS results are reported by the NBS center to the sender of the sample—mostly to maternity wards. The sender of the sample is then responsible to inform the families about the NBS result and the recommendation for confirmatory testing. This is in contrast to other CF-NBS programs, e.g., in Switzerland, where newborns with positive CF-NBS are assigned to a responsible regional CF center and families are directly informed by a CF specialist from this center about the positive CF-NBS result [10]. The G-BA in Germany sets great value on the free choice of the medical center performing confirmatory testing. This leaves the families in the majority of cases alone with the task to arrange an appointment for confirmatory testing. Consequently, this may lead not only to delays in diagnostic work-up but in part also to the situation that positively screened newborns receive diagnostic confirmation in general pediatric hospitals instead of specialized CF centers or even no confirmation at all.

After completion of confirmatory testing, institutions performing diagnostic confirmation are requested to inform the respective NBS centers whether CF has been confirmed or ruled out. This information must be performed in compliance with the strict regulations of German data protection laws and is only possible with written consent of the parents. This flow of information is challenging for all German NBS centers, as currently, a systematic follow-up strategy of all children with positive NBS results—so-called tracking—is not included in the Pediatrics Directive of the G-BA in Germany and is not reimbursed. Consequently, tracking is left to the initiative and expense of the NBS center. This is certainly not only a national phenomenon but will be a challenge for NBS programs in many other countries as the lack of systematic tracking strategies and policies is a shortcoming of most NBS programs worldwide.

In contrast to this, the federal state of Bavaria has established a unique system of centralized organization of confirmatory diagnostics after positive CF-NBS combined with the regular tracking of all positive NBS cases which is financed by the Federal State of Bavaria. Families with positive CF-NBS in Bavaria will be directly informed about the necessary confirmatory testing by a CF specialist from the nearest CF center, if parents agreed to this procedure at the time of consent to NBS [11]. This Bavarian model of information and confirmatory testing, which equals the model performed in Switzerland [10], was only possible because of a previous agreement with all Bavarian institutions involved in confirmatory testing for CF. It is not possible to use the same model in other federal states or by other NBS centers without a previous agreement of all stakeholders involved.

The NBS Center Heidelberg, located in Baden-Württemberg, the neighboring state of Bavaria, started CF-NBS in 2016 according to the G-BA directive without a tracking center. Subsequently, a systematic follow-up of infants tested positive for CF proved very difficult. Therefore, 3 months after the start of CF-NBS, a first tracking strategy was implemented, using repeated written requests to the sender of the NBS sample for the results of confirmatory testing after positive CF-NBS in unresolved cases. Due to an unsatisfactory return rate of confirmatory results also in this system, in February 2018, an intensified second part of the active tracking system by telephone has been introduced in addition to the written requests.

The aim of this study was to evaluate the proportion of results of confirmatory testing of positive CF-NBS cases reported back to the NBS center within the first 24 months after implementation of CF-NBS in Germany for the NBS Center Heidelberg as a model of organization according to the Pediatrics Directive of the G-BA. We assessed the return rate of confirmatory tests over the time course before and after implementation of the first and second stage of the active tracking system and compared the results with those of the scheme in Bavaria. Finally, a cost calculation was performed for a systematic tracking system for the current German NBS including metabolic and endocrine disorders as well as CF.

## Patients and methods

### CF-NBS and organization of confirmatory testing according to the G-BA

**Fig. 1:**
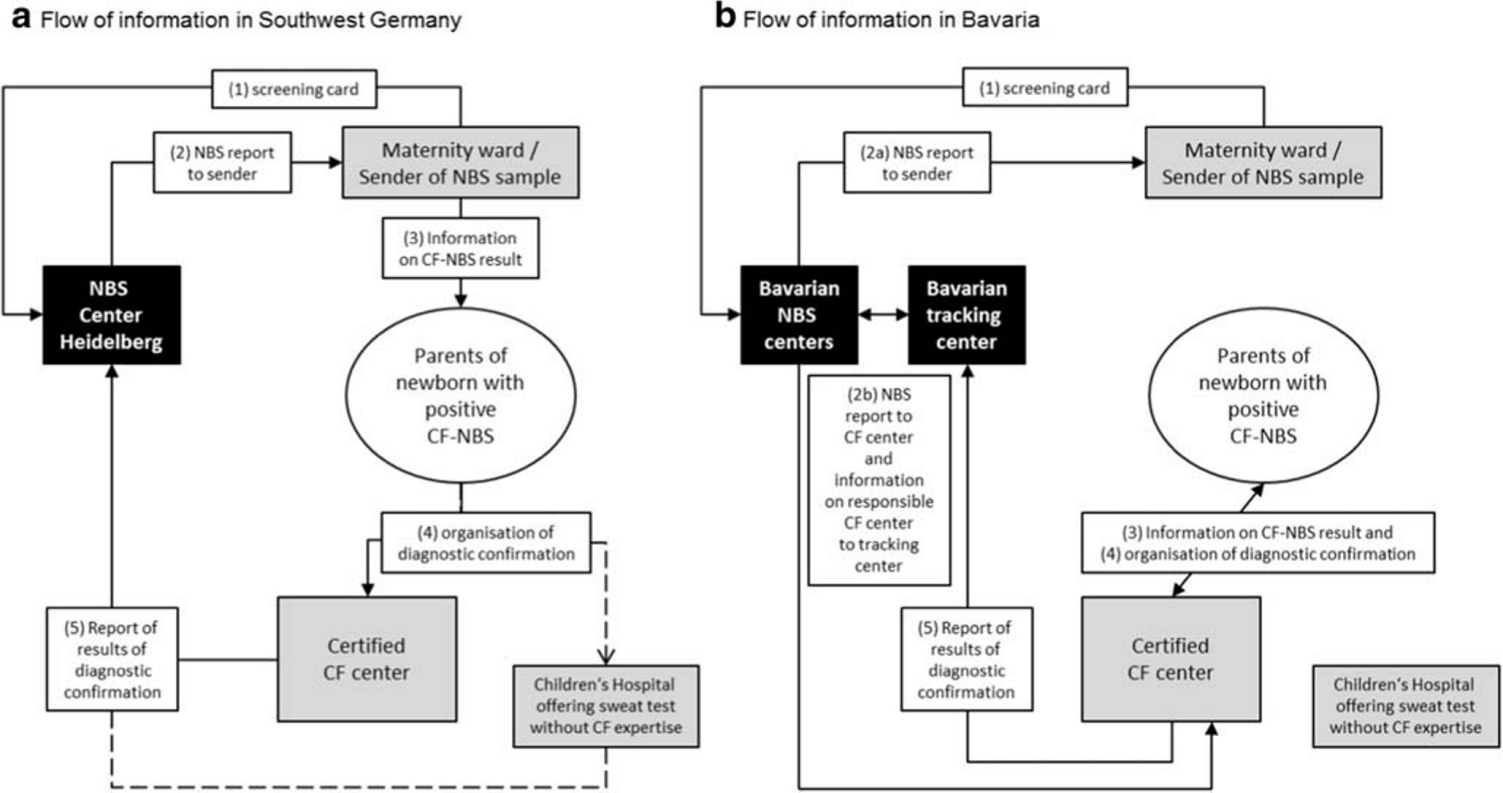
Flow of information and confirmatory testing after positive newborn screening for cystic fibrosis according to regulations of the German Federal Joint Committee (**a**) and in the Bavarian model (**b**).CF Cystic fibrosis; NBS newborn screening

The Heidelberg NBS Center performs NBS for about 140,000 children per year—mainly from south-west Germany—representing about 17% of children born annually in Germany. NBS and communication of abnormal NBS results is performed according to the Pediatrics Directive of the G-BA [5]. According to that, CF-NBS results are primarily transmitted to the sender of the sample as “positive” or “negative” without information about a possible homozygous or heterozygous *CFTR* mutation result. The sender of the sample is then responsible to inform the families about the NBS result and the recommended confirmatory testing. Thereafter, the families themselves—sometimes supported by the sender of the sample—have to contact a regional CF center for confirmatory testing and arrange an appointment for the sweat test. Since the diagnostic confirmation after positive CF-NBS should be carried out in institutions that are experienced in the diagnosis and treatment of CF, the German Cystic Fibrosis Society provides a website with all certified and non-certified CF centers in Germany for better orientation for NBS centers, maternity wards, and parents [12]. After completion of confirmatory testing, the center is requested to report the results to the NBS Center Heidelberg to allow classification of the case as “positive” or “false-positive.” In unresolved cases, the NBS center will try to request the missing information. However, as patients are not primarily assigned to a responsible regional center for confirmatory testing, the NBS Center Heidelberg faces considerable challenges in trying to resolve all screen-positive cases. The flow of information and confirmatory testing after abnormal CF-NBS in this setting is depicted in Fig. 1a.

### Bavarian model of confirmatory testing and tracking

In the federal state of Bavaria, a tracking center for all NBS cases has been successfully in operation since 1999, receiving all results from the two Bavarian NBS laboratories on a daily basis. In addition, there is a cross check with the residents’ registration offices. The Bavarian tracking center—by approval of federal state legislation—has access to the federal state’s birth registry and can therefore assure completeness of NBS. Families will be contacted by the tracking center if data matching with the birth registry shows absence of NBS to inquire if the family did actually not want to have NBS performed for their child or whether, e.g., the sample has been lost. In contrast to the other German federal states, parents in Bavaria have the opportunity to agree at the time of consent to NBS, that they will be directly informed about a positive CF-NBS result by a CF specialist from the nearest CF center [11]. This procedure, depicted in Fig. 1b, allows direct contact with the CF center to complete information on confirmation of unresolved cases. Usually, the CF center documents the results of confirmatory testing on a one-page form and sends the data to the tracking center with parental consent. If no information about confirmatory testing is sent to the Bavarian tracking center within 35 days, the CF center will be contacted by telephone. Of note, in this model, the parents still have the possibility to choose another CF center by themselves, although they are contacted primarily by one CF center. Therefore, also in this system, there is no violation of families’ free choice of medical center.

### Tracking system established in the NBS Center Heidelberg

**Fig. 2:**
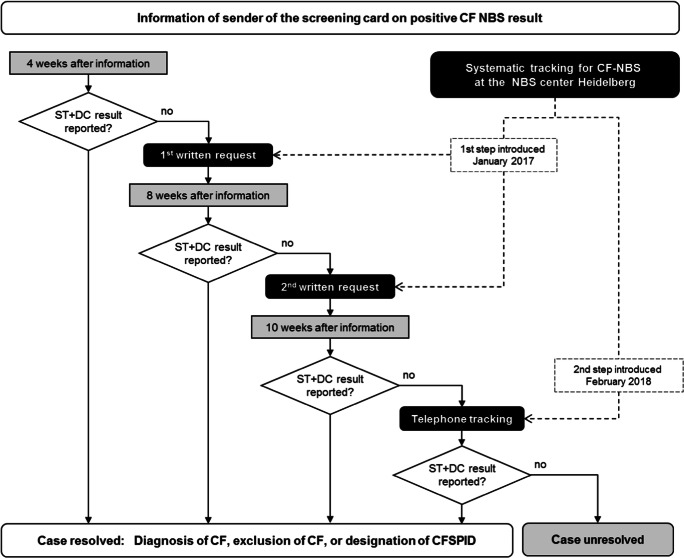
Tracking system established at the Heidelberg NBS Center after abnormal CF-NBS. CF cystic fibrosis, NBS newborn screening, CFSPID cystic fibrosis screen positive, inconclusive diagnosis, DC diagnostic confirmation, ST sweat test

CF-NBS as part of the German NBS panel started at the Heidelberg NBS Center in October 2016. Before this time point, CF-NBS had already been performed in the context of a pilot study [8]. After 3 months, starting in January 2017, a systematic tracking strategy was implemented using written requests to the sender of the NBS sample for the results of confirmatory testing in unresolved cases. These requests were sent automatically 4 weeks and again 8 weeks after information about a positive CF-NBS result (compare Fig. 2).

Due to an unsatisfactory return rate of confirmatory results of only 43% also in this system, in February 2018, an additional active tracking system by telephone was added. For this system, a medical documentation officer (CL) was trained by the responsible physician for NBS (GG) to perform standardized telephone interviews with the sender of the initial NBS sample and, if required, also with the institution performing confirmatory testing after abnormal CF-NBS. This strategy was performed in addition to written requests if cases had remained unresolved 10 weeks after information of a positive CF-NBS result (compare Fig. 2). Tracking by telephone was carried out for new unresolved cases, but also retroactively for all previous cases that were still unresolved despite written requests.

### Data collection

Primary data for CF-NBS were prospectively documented and stored in the IT system of the NBS Center Heidelberg. Here, also the suspected diagnosis of CF was documented for cases with positive CF-NBS, and the final diagnosis (CF, CFSPID (CF screen positive, inconclusive diagnosis), or false positive) was noted after confirmatory results had been returned. Written requests were automatically generated in the NBS IT system in positive CF-NBS cases if a final diagnosis had not been reported and documented 4 weeks (first written request) and again 8 weeks (second written request) after first NBS report (compare Fig. 2). For additional tracking and documentation of NBS, all cases with abnormal NBS results (recalls) were transferred to an Access database. For the retrospective telephone tracking, all cases still unresolved were extracted to an Excel file for tracking to be performed and documented by the documentation officer. Accordingly, in the prospective telephone-tracking cohort, all cases still unresolved 10 weeks after first NBS report were extracted for telephone tracking. Information on the tracking progress and reported results were prospectively documented in Excel, Access database, and the NBS IT system.

### Informed consent

When consenting to performance of NBS, legal guardians were also asked to provide written informed consent for tracking in case of abnormal NBS results in their child. Evaluation of the effect of tracking was performed as part of quality assurance for the NBS laboratory Heidelberg. Ethical approval for evaluation of CF-NBS at the NBS Center Heidelberg in comparison with results from the Bavarian NBS was obtained from the institutional review board of the University Hospital Heidelberg in the context of a research project evaluating parents’ perspectives after positive CF-NBS (Number S-718/2017).

### Cost calculation

To assess the health economic aspects of implementing a tracking system for CF-NBS, a cost calculation was performed including required personnel and resources. As writing and handling of written requests in unresolved cases after positive NBS, tracking letters, telephone calls etc. are not performed by separate personnel for metabolic, endocrine disorders, or CF, this calculation was performed for a tracking concept for all target disorders of the German NBS panel at the time of evaluation [13]. Evaluation of required staff time was performed according to experience from the study period at the NBS Center Heidelberg. In addition, staff requirements were compared with personnel required in the tracking system at the Bavarian tracking center.

## Results

Over the first 24 months after implementation of CF-NBS into routine NBS (October 2016 until September 2018), 282,353 newborns were screened for metabolic and endocrine disorders according to the German NBS panel at the NBS Center Heidelberg. For 281,907 of these children (99.8%), also CF-NBS was performed. For the remaining 446 children (0.2%), CF-NBS was declined by the parents. During the period of 24 months, an abnormal CF-NBS result was detected in 244 children (0.09%). The reason for this recall rate slightly below 0.1% despite the SN strategy described in Supplementary Figure 1 is that in cases with IRT > 99.9 percentile, repeat measurements from several areas of the filter paper card are performed. The SN strategy is only followed in our lab if the repeat measurements confirm IRT > 99.9 percentile.

In cases with positive CF-NBS, the senders of the respective screening card were informed about the result and the recommendation for confirmatory testing. However, the result of confirmatory testing was not reported to the NBS Center for more than half of all infants with positive CF-NBS. Prior to the implementation of the active telephone tracking in February 2018, the percentage of unresolved cases after abnormal CF-NBS was still 57% despite written requests. The consecutive telephone tracking led to a continuous improvement in the rate of resolved cases over time. After 13 months of telephone tracking for all unresolved cases from October 2016 until September 2018, the rate of children with returned information on confirmatory testing (evaluated up to March 2019) could be raised to 84%. Tracking by telephone performed retroactively for all cases from 2016 and 2017 that were still unresolved at the NBS center despite written requests allowed to solve 58 cases from this time period—of these, 19 with a confirmed diagnosis of CF. In all CF patients from this group with information on the date of diagnosis available (16 of 19), CF diagnosis had already been established before telephone tracking. However, two children in this group had a rather late diagnosis at age 3 and 5 months. It can be postulated that in these cases, diagnostic confirmation was started only after the second written request from the NBS center.

Altogether, 141 children (58%) were classed as NBS false positive. In 22% of children, the diagnosis of CF was confirmed (*n*=54), and one child was classified as CFSPID [14]. In 3.7% of cases, the child had deceased (*n*=7), no confirmatory testing was performed due to a palliative situation (*n*=1), or information was missing because the NBS sample had been sent from abroad (*n*=1). For 16% of all children with abnormal CF-NBS, there was still no information on the result of confirmatory testing available despite systematic tracking.

**Fig. 3:**
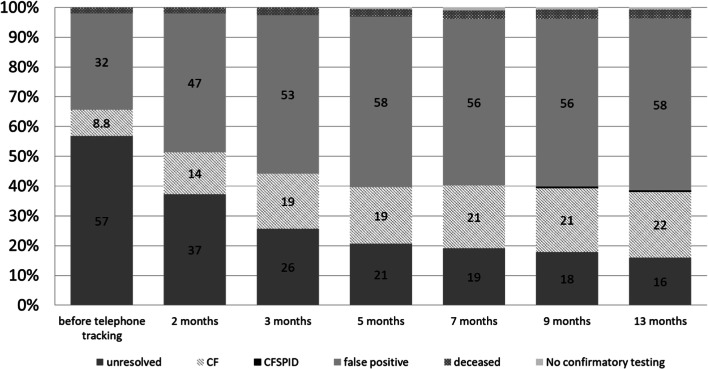
Effect of telephone tracking on rate of resolved cases after positive newborn screening for cystic fibrosis. Proportion of children (in %) with different results following positive CF-NBS with respect to the total number of children with positive CF-NBS results (in %) at the respective time points of the evaluation. CF cystic fibrosis, NBS newborn screening, CFSPID cystic fibrosis screen positive, inconclusive diagnosis

Figure 3 shows the proportion of the different results after diagnostic confirmation following positive CF-NBS with respect to the total number of children with positive CF-NBS results at the respective time points before and after introduction of a systematic telephone tracking at the NBS Center Heidelberg.

### Results from the Bavarian tracking center

For CF-NBS, the Bavarian tracking center reports a rate of resolved cases after positive CF-NBS of 98% for the year 2016, 99% for 2017, and 100% for 2018.

**Fig. 4:**
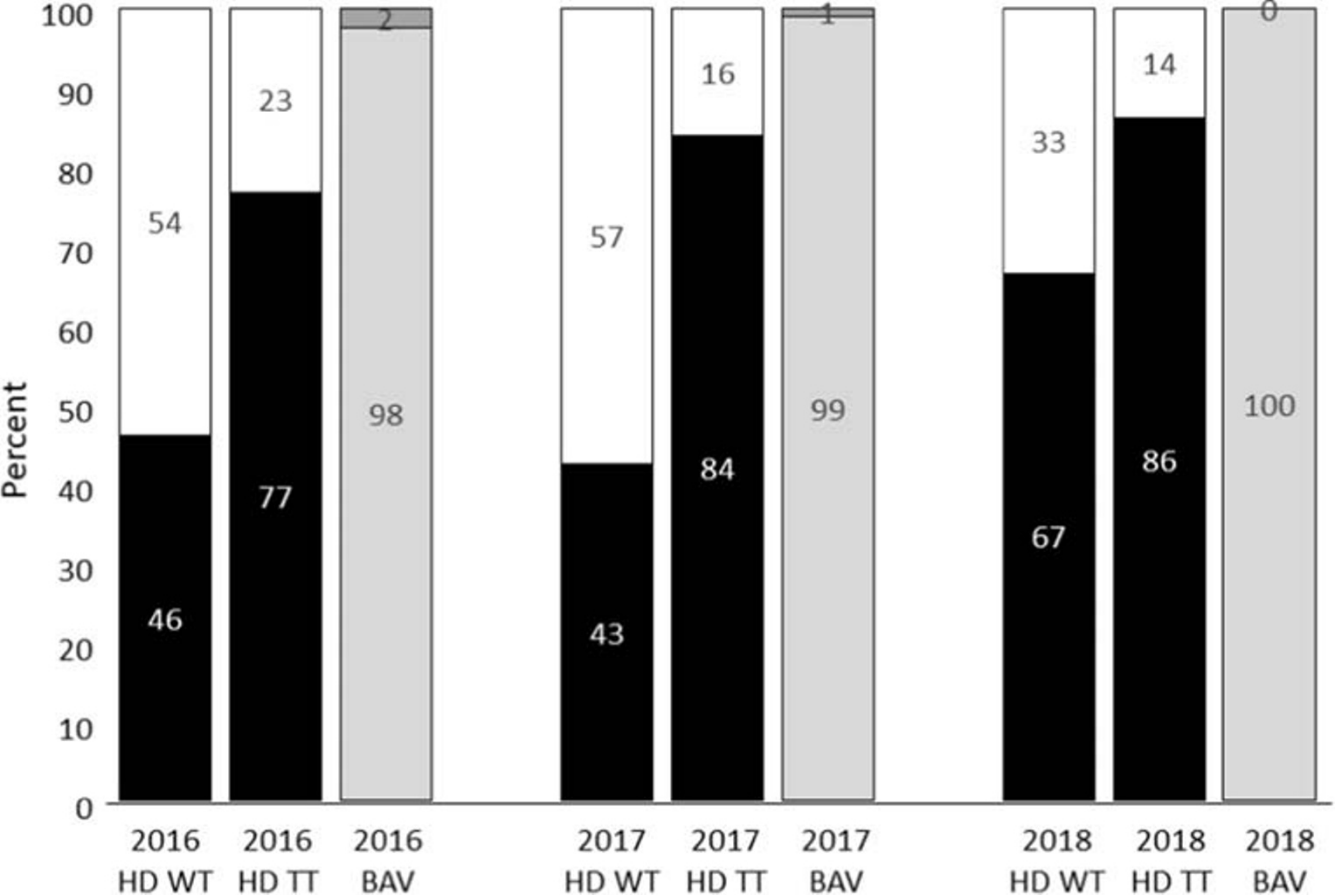
Resolved cases (in %) per year 13 months before and after application of the active telephone tracking for CF-NBS (HD) in comparison with resolved cases in Bavaria (BAV) where a centralized tracking center for all NBS cases is in place. (Abbreviations: HD WT Heidelberg with written tracking only, HD TT Heidelberg with telephone tracking, BAV Bavaria, CF cystic fibrosis, NBS newborn screening, BAV Bavaria; HD Heidelberg, TT telephone tracking, WT written tracking only

When comparing the rate of resolved and unresolved cases over the three calendar years 2016, 2017, and 2018 at the Heidelberg NBS Center, an improvement in the rate of resolved cases could be documented over the course of time. For cases from the year 2016, telephone tracking led to an increase of resolved cases from 46 to 77% (Pearson’s chi-squared test with Yates’ continuity correction *p* = 0.37), for cases from the year 2017 from 43 to 84% (*p* = 0.002), and for cases from the year 2018 from 77 to 86% (*p* = 0.32). But compared with the values achieved in Bavaria, there is still a significant difference in every year (2016: *p* = 0.014; 2017: *p* = 0.0003; 2018: *p* = 0.0001; Pearson’s chi-squared test with Yates’ continuity correction) even after implementation of a tracking system including written requests and telephone tracking (Fig. 4).

### Cost calculation for a tracking system for newborn screening for metabolic and endocrine disorders and CF

A tracking concept for all target disorders of the German NBS panel at the time of evaluation (16 disorders: 2 endocrine, 13 metabolic disorders, CF) would require the following personnel. Two secretary staff members (each 50% part-time share to assure a substitute during vacation and illness) are required for identification of unresolved cases in the NBS IT system, verification of unresolved status after review of repeat dried blood samples in the laboratory system, and availability of returned reports of confirmatory testing. In cases with unresolved status after internal review—in ambiguous cases after consultation with the responsible physician of the NBS center—preparation and sending of written requests is performed by the secretary staff. A medical documentation officer (40% part-time share) is required for documentation of all returned results in a tracking database. In addition, telephone tracking is performed by both secretarial staff and documentation officer in cases remaining unresolved despite sending of written requests. This includes telephone calls with birth hospitals, pediatricians, and centers performing confirmatory testing for metabolic/endocrine disorders and CF. Contact with these centers is only possible under the prerequisite that information on the responsible center is available at the NBS center. A physician (75% part-time share) is required for medical evaluation of returned results and decision on the final diagnosis—e.g., confirmed, false positive, still unresolved—and requirement for further information and tracking. The physician is also responsible for all medical requests from senders, pediatricians, and centers performing confirmatory testing, as well as for advice on confirmatory testing and requests from families concerning medical aspects. The physician has final responsibility for validation of all cases after abnormal NBS. An IT specialist is required for programming the tracking database and transfer of results from the NBS IT system into the tracking structure. After programming and implementation of the required IT infrastructure, IT has to be available 5 days per week—the time share for the tracking system being about 10% of one person. In addition, a lump sum for general infrastructure is required and is estimated at 10,000 € (about US$11,080) per year. With respect to current cost of personnel (for details on cost calculation, compare Supplementary Table 1) and given the number of about 140,000 children screened at the Heidelberg NBS Center, this results in additional NBS cost for a systematic tracking system of 1.20€ (about US$1.40) per child including telephone tracking. This is comparable with the cost per child for tracking in Bavaria. The Bavarian tracking system requires 1.51€ per child for the follow-up system and 0.25€ for assurance of completeness, resulting in a total cost of 1.76€ per child (about US$2.0) in Bavaria.

## Discussion

Newborn screening has a large impact on children and their families, as well as on society, especially on health care systems and social systems. The success of every screening program depends on the successful and preferably complete inclusion of the population targeted and on the adequate, timely, and competent clarification of all abnormal screening results. This assures that affected individuals are treated in a timely manner and can benefit from early detection.

CF-NBS pilot studies in Germany found a prevalence of CF of 1 in about 4800 newborns [8]. Data from the presented evaluation for one of the largest German NBS centers and from the national NBS report for Germany [7] document that a considerable number of cases after positive CF-NBS remain unresolved. This is most certainly not a german phenomenon, but a challenge for most NBS programs—especially when confirmatory testing is not organized and monitored centrally until all cases have been resolved.

For Germany, NBS programs of only three federal states (Bavaria, Hessen, and Saxony-Anhalt) feature a tracking center. In Bavaria, with parents’ previous consent, a specialist will inform the families about the positive NBS result and necessary confirmatory testing. Together with this first provision of competent information, a prompt appointment for sweat testing will be offered. The tracking center will get information for clarification of each case directly from the responsible clinical center [11] and will contact this center directly to complete information on unresolved cases. In addition, the tracking center does assure the completeness of requested repeat samples and of confirmatory testing [15]. If the result of confirmatory diagnostics has not been transmitted to the tracking center after a set time period, the center responsible for confirmatory testing will be contacted by telephone.

In contrast, according to the national regulation in the other German states, the sender of the NBS sample is responsible for communication of abnormal NBS results to the families. No regulation is in place regarding systematic tracking and responsibilities for reminding the parents if no confirmatory results are returned to the NBS center.

Data from the tracking center in Bavaria demonstrate that a reminder for repeat sampling or confirmatory testing is required in about 18% of cases with abnormal NBS results for endocrine or metabolic disorders, and even in about 40% of cases after abnormal hearing screening [15–17]. For CF-NBS, the Bavarian system of information, confirmatory testing, and tracking results in an almost 100% rate of resolved cases. In all federal states without a tracking center, the documentation and follow-up after abnormal NBS results has to be performed by the NBS center alone, which affects effectiveness. The introduction of CF-NBS into routine NBS in Germany in the year 2016 posed a particular challenge for follow-up of results, as confirmatory testing was not restricted to certified CF centers by the G-BA, and sweat testing is offered by a large number of pediatric hospitals and centers [12]. This differs from confirmatory testing after abnormal NBS for metabolic disorders, which is performed in a small number of specialized metabolic centers. For tracking of results after abnormal CF-NBS, exact information on the responsible institution performing the sweat test is required. Due to the larger number of institutions offering confirmatory testing for CF, it is not possible to conclude the institution or center performing the sweat test from the families’ place of residence.

NBS provides a large individual benefit for affected children and their families due to the possibility of preventive treatments. These measures are of high benefit from a health political as well as health economic perspective [18, 19], given the negative impact of late diagnosis and treatment for the individual patient and the society. If abnormal NBS results are not followed by adequate confirmatory testing, affected individuals will not benefit from early identification. Therefore, this scenario is not superior to a scenario without NBS for this condition while the health system is still covering the costs for NBS.

The presented evaluation demonstrates that in CF-NBS without an active telephone tracking in place, more than half of all abnormal NBS results (57%) remain unresolved for the NBS center due to lack of information on the result of confirmatory testing. One possible scenario in these cases is that the recommended confirmatory testing has not been performed. This could be due to deficits in communication of the NBS result to the family, families’ reluctance against confirmatory testing, or difficulties for families in organizing an appointment at a specialized center. Sometimes, also repeated difficulties in performing the sweat test in newborns due to insufficient sweat sample volume can lead to families’ withdrawal from the confirmatory process. In any of these scenarios, the child affected by CF will remain undiagnosed and cannot benefit from early therapeutic interventions, with costs for NBS not resulting in any benefit for the child, family, or society. If a child with abnormal CF-NBS but unaffected by CF (false-positive NBS) does not undergo confirmatory testing, there will be no negative health consequences for this child. However, the lack of final information about the normal health status of their child could result in continuing increased anxiety for parents if they had been informed about the abnormal CF-NBS but did not receive confirmation that their child is unaffected by CF. This anxiety has been documented in studies on communication of false-positive NBS results for metabolic disorders in parents informed insufficiently about false-positive NBS findings [20].

A second scenario in unresolved cases could be that confirmatory diagnostics has been performed, but the results have not been communicated to the NBS center. This could affect children with confirmed CF as well as children with false-positive CF-NBS. In these cases, affected children will have benefitted from early diagnostics, and parents of both affected and unaffected children should have received adequate information and counseling. However, the lack of returned results of confirmatory testing to the NBS center has negative implications for the NBS program. Without complete information on confirmed and false-positive cases after abnormal CF-NBS, the NBS centers cannot provide sufficient information on true-positive and false-positive cases and therefore on the positive predictive value of the current CF-NBS strategy. These quality parameters are however crucial for a successful quality management and continuous quality improvement in NBS.

After 13 months of systematic tracking in CF-NBS including active telephone tracking, the rate of unresolved cases could be reduced from initially 57 to 16%. This demonstrates that in a NBS program, a tracking system is crucial to receive the information on final diagnosis essential for quality assurance—at least in the majority of cases. However, the comparison to the Bavarian model shows that the effect of a tracking system remains limited by the absence of a centralized organization of confirmatory testing and tracking. It has to be noted as a limitation to this comparison that the centralized organization of confirmatory diagnostics and tracking for one federal state has additional advantages, e.g., concerning authorization and arrangements with CF centers.

During the 24 months evaluated here and given the incidence of CF established in CF-NBS pilot studies in Germany [8], 56 children affected by CF would have been expected in a cohort of 281,907 children screened. Under consideration of the first national report on CF-NBS in Germany for the year 2017, reporting a final diagnosis of CF or CFSPID for 21% of children with abnormal CF-NBS [7], 52 children from the 244 children with abnormal CF-NBS from the NBS Center Heidelberg would be expected to be confirmed with CF or CFSPID. Therefore, it can be assumed that all or almost all truly affected children have been reported back to our center, as under implementation of the tracking system, 54 cases of CF and 1 case of CFSPID have been reported as confirmed. The unresolved cases will therefore presumably include mostly false-positive cases of CF-NBS.

For 21 of 39 children (54%) for whom despite tracking no result of confirmatory testing was available at the end of the reported period, the sender of the NBS sample stated that results of confirmatory testing could not be transmitted to the NBS center due to data protection regulations as no repeated consent of families for this procedure was available. This was stated by the senders despite the fact that all parents had already consented to data transfer to the NBS center in case of confirmatory testing when consenting for NBS. As a consequence, the tracking strategy in these cases is now complemented by letters from the Heidelberg NBS Center to the parents asking them directly to transmit or initiate transmission of confirmatory results to the NBS center.

The first national screening report for Germany after implementation of CF-NBS into the national NBS panel for the year 2017 demonstrated that the problem with unresolved cases or insufficient information to the NBS center is not an issue of a single NBS center but a national problem. For the whole of Germany, 24% of all cases with abnormal CF-NBS remained unresolved at the NBS centers due to lack of information on confirmatory testing [7]. In addition to this obvious deficit in CF-NBS, the same report shows that also in NBS for metabolic or endocrine disorders, the lack of a systematic tracking structure results in a rate of 17% unresolved cases after abnormal NBS [7]. The national screening report also clearly demonstrates the effect of a tracking center on the rate of resolved NBS cases: for one NBS center performing NBS in one federal state in collaboration with a tracking center, the rate of unresolved cases in this setting becomes < 1%. For the same NBS center, in an area without an associated tracking center, the rate of unresolved cases after abnormal NBS is 9.5% [7].

Given the negative consequences of unresolved or unreported cases on the success of a NBS program, it would be highly desirable that a revision of the Pediatrics Directive concerning CF-NBS is considered. This revision should include the opportunity to be informed by a specialist about the necessary confirmatory testing after positive CF-NBS identical to the Bavarian model. In addition, given the increasing number and complexity of target disorders in the German and other NBS programs, it appears essential to include a tracking system and to reimburse the cost for this important task as part of a NBS program. Hopefully, these issues will be addressed in the pending re-evaluation of CF-NBS by the G-BA after the first years after introduction into the German NBS panel. The results from our study are also of relevance for NBS programs in many other countries as the lack of systematic tracking strategies and policies is a shortcoming of most NBS programs worldwide.

## Conclusion

The success of every screening program depends on the successful and preferably complete inclusion of the population targeted and on the adequate, timely, and competent clarification of all abnormal screening results. The implementation of a tracking system achieves a distinct improvement of clarified cases in the German CF-NBS system. However, the effect of a tracking system is limited if children with positive NBS are not directly assigned to a responsible center for confirmatory testing. These aspects should be included in a possible revision of the current organization of CF-NBS in Germany and should be considered when setting up CF-NBS programs in other countries.

## Electronic supplementary material

ESM 1 (DOCX 62 kb)

## Abbreviations

CF: Cystic fibrosis
CF-NBS: Newborn screening for cystic fibrosis
G-BA: Gemeinsamer Bundesausschuss
IRT: Immunoreactive trypsin
NBS: Newborn screening
PAP: Pancreatitis-associated protein
SN: Safety net strategy
