# Supplementary material for: Implementing a tracking system for confirmatory diagnostic results after positive newborn screening for cystic fibrosis—implications for process quality and patient care
Source: Eur J Pediatr. 2020 Oct 26;180(4):1145–55. doi: 10.1007/s00431-020-03849-4 (PMC7940155; doi:10.1007/s00431-020-03849-4)
Supplement: Supplementary file 1 — (DOCX 62 kb) [file 431_2020_3849_MOESM1_ESM.docx]

**Supplementary Figure 1**

CF- NBS protocol with safety net currently used for the nationwide CF-NBS program in Germany.


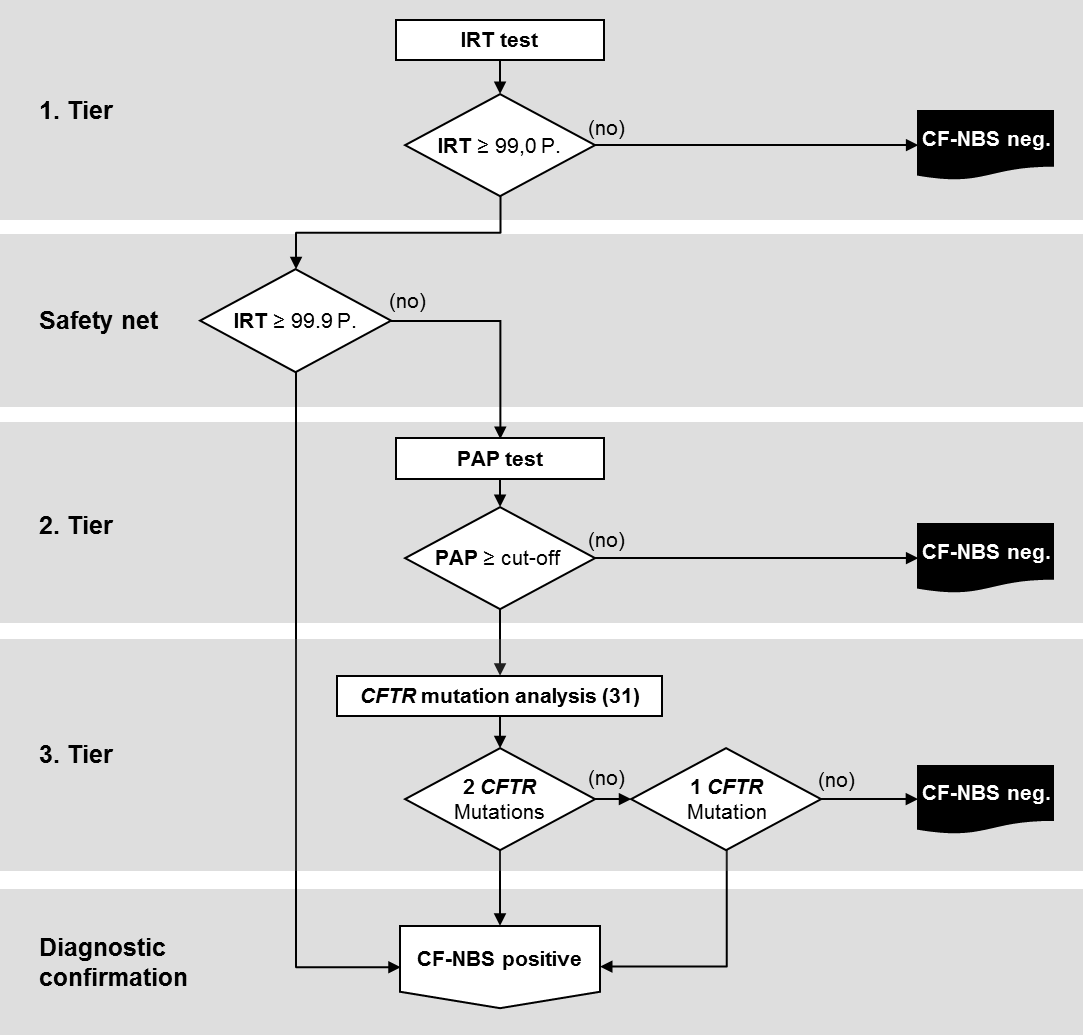


Figure legend

CF = Cystic fibrosis; NBS = newborn screening; CFTR = Cystic Fibrosis Transmembrane Conductance Regulator; IRT = Immunoreactive trypsine; PAP = Pancreatitis-associated protein; P. = Percentile; neg. = negative

**Supplementary Table 1Cost calculation for a tracking concept of newborn screening for metabolic disorders, endocrine disorders, and cystic fibrosis**

| Personnel | Wage group | Salary/year for full-time position  in € | Part-time  share | Cost for tracking center  in € |  |
| --- | --- | --- | --- | --- | --- |
|  |  |  |  |  |  |
| Physician | TV-Ä2 | 109,100 | 0.75 | 81,825 |  |
| Secretary staff | TV-UK5 | 49,000 | 2 x 0.5 | 49,000 |  |
| Documentation officer | TV-UK8 | 55,000 | 0.4 | 22,000 |  |
| IT specialist | TV-UK11 | 75,000 | 0.1 | 7,500 |  |
|  |  |  |  |  |  |
| Lump sum for general infrastructure |  |  |  | 10,000 |  |
|  |  |  |  |  |  |
| SUM |  |  |  | 170,325 |  |
|  |  |  |  |  |  |
| **Cost per newborn screened** |  |  |  |  | **1.20 €** |

Cost of personnel is based on salaries according to wage agreements in Germany as of 2019

**Abbreviations**

TV-UK Wage agreement for non-medical staff at university hospitals

TV-Ä Wage agreement for physicians at university hospitals

€ Euros
